# Survival After a Transcranial Bihemispheric Stabbing with a Knife: Case Report and Literature Review

**DOI:** 10.7759/cureus.5512

**Published:** 2019-08-29

**Authors:** Peter A Ebeling, Abdullah N Ghali, Ryan McDermott, Jean-Louis R Caron, Daniel L Dent

**Affiliations:** 1 Surgery, University of Texas Health Science Center at San Antonio, San Antonio, USA; 2 Neurosurgery, University of Texas Health Science Center at San Antonio, San Antonio, USA

**Keywords:** penetrating brain injury, low velocity penetrating brain injury, non-missile brain injury, bihemispheric injury, transcranial stab wound

## Abstract

Low-velocity penetrating brain injuries (PBIs), also referred to as nonmissile brain injuries, typically result from stabbings, industrial or home accidents, or suicide attempts. A great deal of literature has focused on the injury patterns and management strategies of high-velocity PBIs. However, there are substantially fewer large, contemporary studies focused solely on low-velocity PBIs. Here, we present an interesting and uncommon case of a patient who suffered a bihemispheric stab wound involving the basal ganglia. A 22-year-old man presented to the hospital with a stab wound to the left calvarium. His initial Glasgow Coma Scale (GCS) score was 13, but he rapidly declined to a six and was intubated. He was emergently taken to the operating room for craniectomy, knife removal, and external ventricular drain placement. On the first postoperative day, the patient was following commands with all extremities. He was discharged to a rehabilitation facility 13 days postinjury. One year after the injury, the patient was free of major neurologic sequelae. This report illustrates a rare case of a good functional outcome after a transcranial stabbing with multiple imaging and exam findings usually associated with poor outcomes.

## Introduction

Penetrating brain injuries (PBIs) are usually classified according to velocity or injury pattern. Low-velocity PBIs, also referred to as nonmissile brain injuries, typically result from stabbings, industrial or home accidents, or suicide attempts [1]. Although generally considered to be less traumatic than high-velocity injuries, such as gun shot wounds (GSWs), this mechanism may result in substantial intracranial parenchymal or vascular trauma. A great deal of literature has focused on the injury patterns and management strategies for high-velocity PBIs. However, there are substantially fewer large, contemporary studies focused solely on low-velocity PBIs. These injuries present significant challenges to medical providers due to their relative rarity and the patients’ complex care needs. Here, we present an interesting and uncommon case of a patient who suffered a bihemispheric stab wound with subcortical structure injuries and had a favorable outcome.

## Case presentation

**Figure 1:**
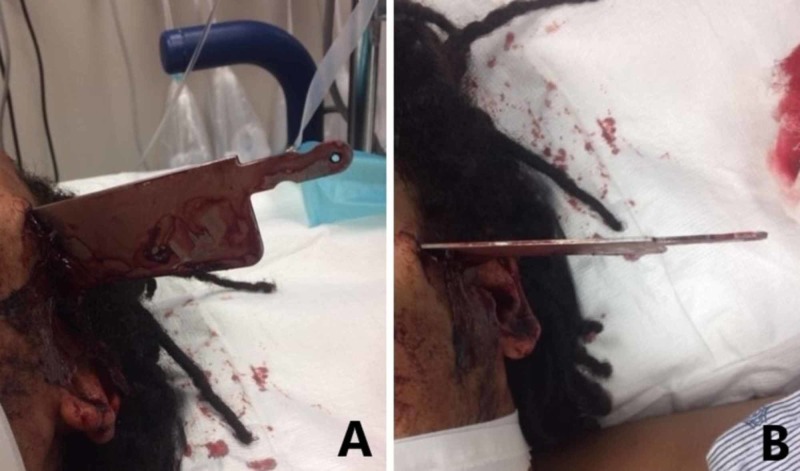
The patient presented to the emergency room with a knife penetrating the calvarium in the left temporal area. (A) View of the injury from inferior to the mandible. (B) Anterior view of the injury.

A 22-year-old man was brought to a level one trauma center by emergency medical services from the scene of a stabbing. Emergency medical personnel reported the patient was found in the field with a large kitchen knife protruding from the left side of his head (Figure 1). Upon arrival, the patient was awake, alert, and moving all extremities. His initial Glasgow Coma Scale (GCS) score was 13. However, he subsequently became combative, declined to a GCS of six, and was intubated for airway protection. 

**Figure 2:**
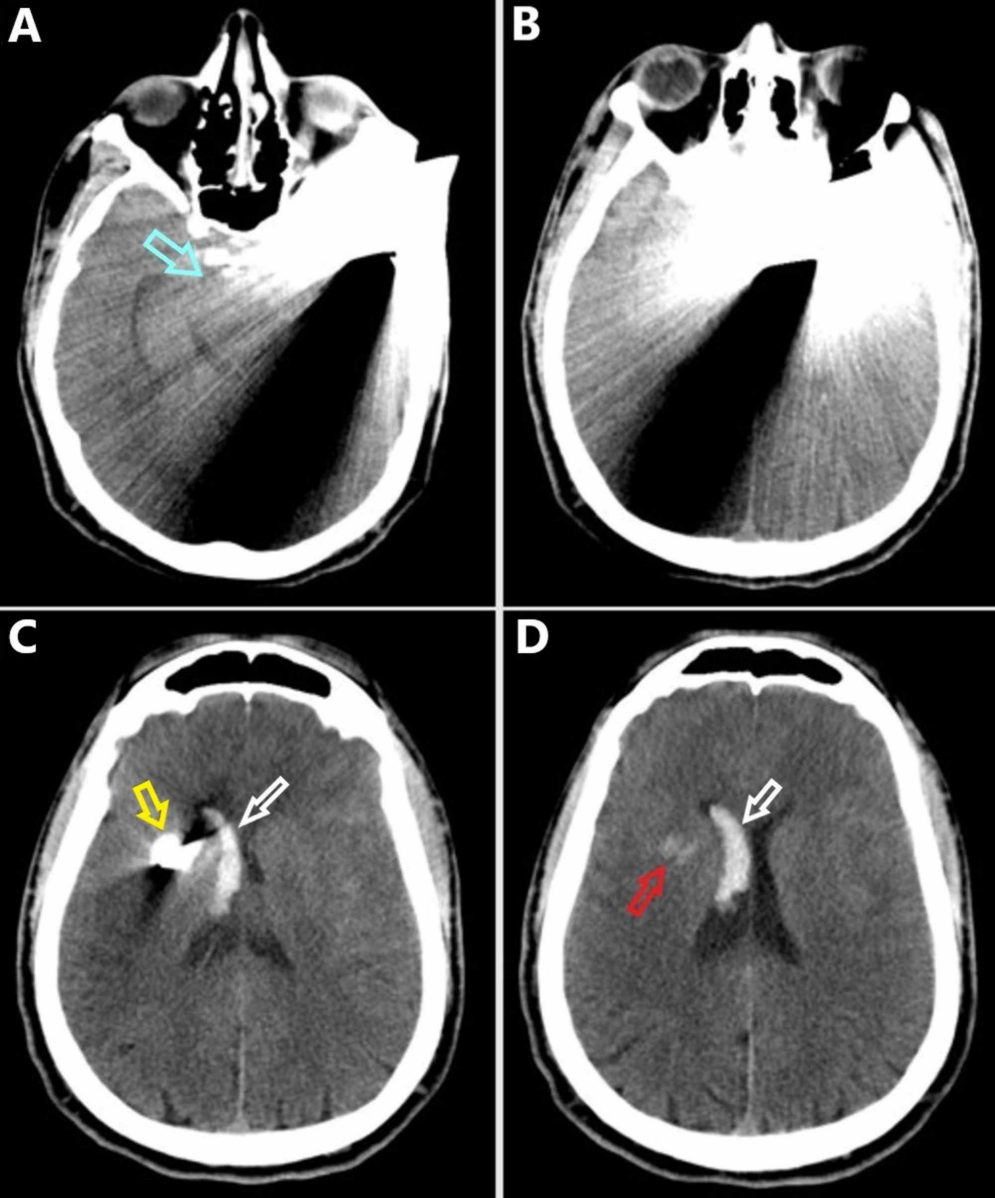
Noncontrasted preoperative CT scan of the head in the axial plane progressing from inferior (A) to superior (D). (A) The knife penetrates the calvarium in the left temporal region. Note the midbrain is in close proximity to the injury tract (blue arrow). (B) The knife begins to penetrate across midline. (C) The tip of the knife is evident in the right hemisphere (yellow arrow). Note the intraventricular hemorrhage in the right lateral ventricle frontal horn and body (white arrow). (D) There is a small intraparenchymal hemorrhage in the right basal ganglia (red arrow). Intraventricular hemorrhage is evident within the right lateral ventricle frontal horn and body (white arrow).

Primary and secondary surveys did not reveal additional injuries. Physical exam was significant for a large knife protruding from the left cranium approximately 10 cm. Medical history was obtained from the next of kin, which was only remarkable for illicit drug use. Laboratory investigations were remarkable for a leukocytosis of 13 K/µL and a lactate of 5.8 mmol/L. The patient was taken for noncontrasted head CT exam, which showed a moderate right lateral intraventricular hemorrhage, a small intraparenchymal hemorrhage in the right basal ganglia, a 3 mm subdural hematoma along the left cerebral convexity, and a small subarachnoid hemorrhage in the basal cisterns (Figure 2).

**Figure 3:**
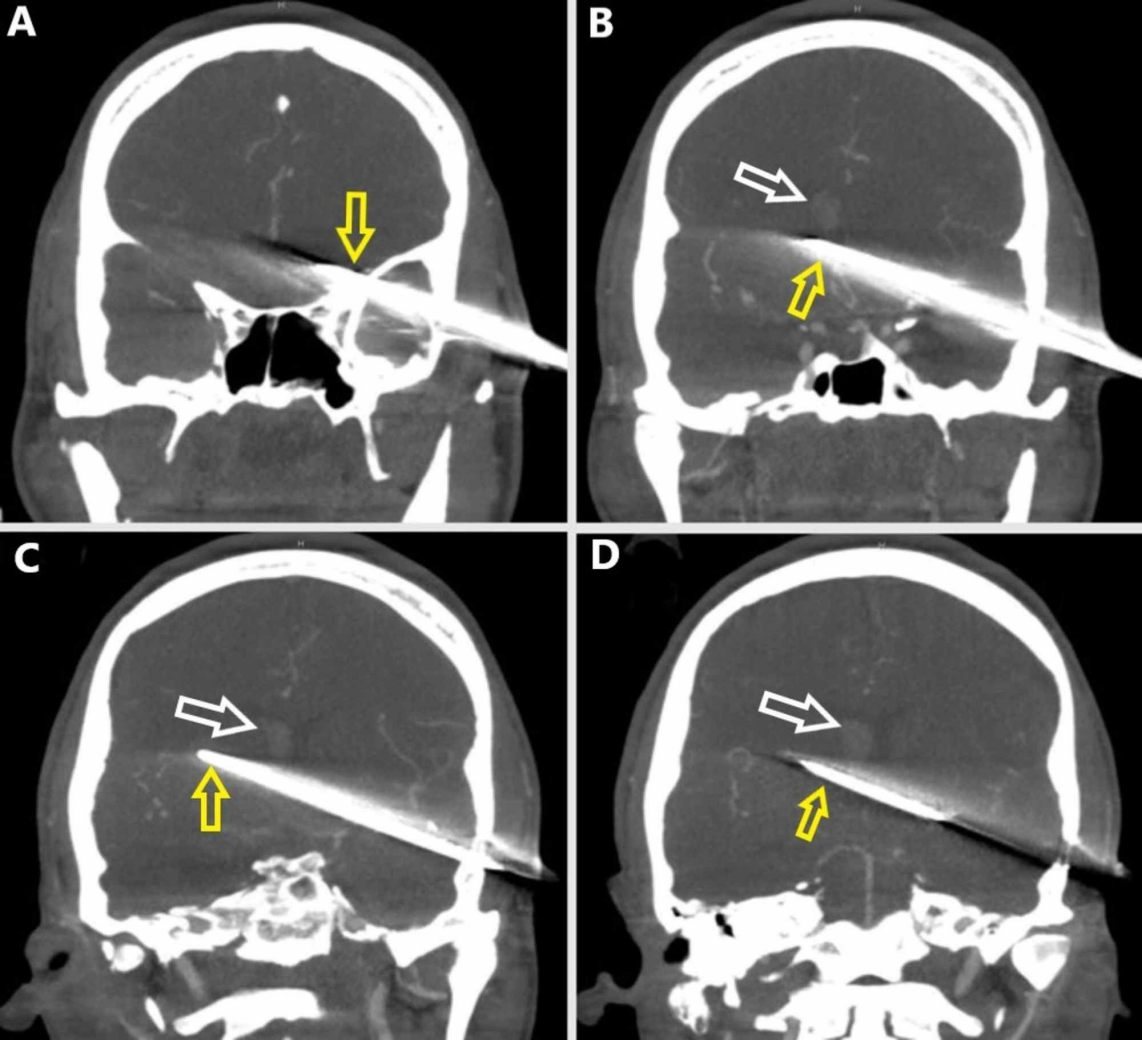
Coronal plane preoperative CT angiography in the vascular window setting progressing from anterior (A) to posterior (D). (A) The knife (yellow arrow) penetrates the left temporal bone. (B) The knife (yellow arrow) passes midline, and a right lateral intraventricular hemorrhage is evident (white arrow). (C) The knife is seen along the full extent of its intracranial route (yellow arrow). The right lateral intraventricular hemorrhage is evident (white arrow). (D) Posterior limit of the intracranial knife (yellow arrow). The right lateral intraventricular hemorrhage is evident (white arrow).

There was approximately 10 cm of intracranial penetration projecting from the left temporal bone through the left basal ganglia and ultimately terminating in the right basal ganglia. Subsequent CT angiogram of the brain did not show a definitive vascular injury, although there was significant beam artifact from the knife (Figure 3). 

**Figure 4:**
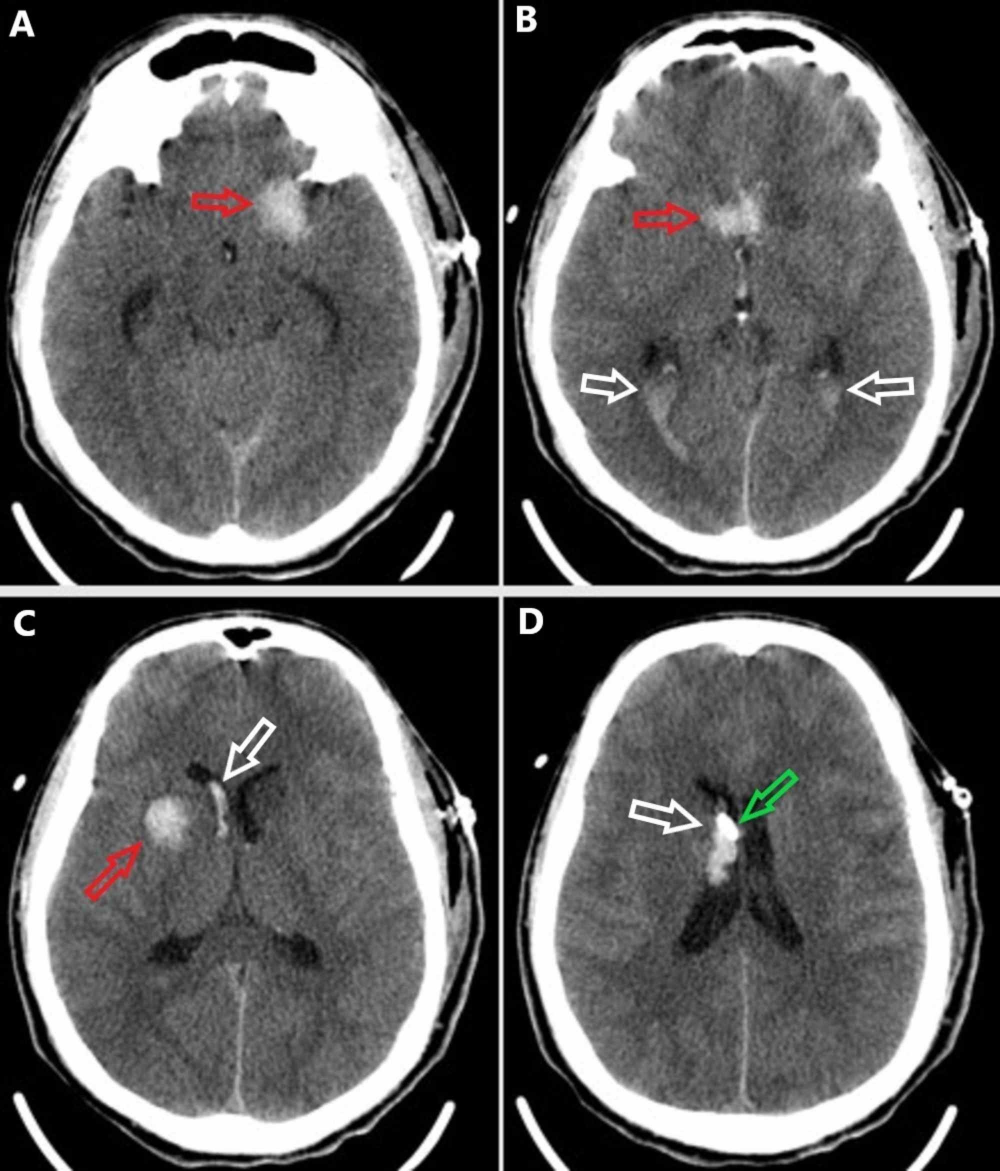
Postoperative noncontrasted head CT scan in the axial plane progressing from inferior (A) to superior (D). (A) There is an intraparenchymal hemorrhage in the left anterior temporal lobe along the injury tract (red arrow). (B) The intraventricular blood is seen layering into the right occipital horn of the lateral ventricle and the bilateral atria (white arrows). Injury tract hemorrhage is seen in the midline (red arrow). (C) There is a tract hemorrhage within the right basal ganglia (red arrow) and intraventricular hemorrhage within the right lateral ventricle frontal horn and body (white arrow). (D) The external ventricular drain tip (green arrow) is visible adjacent to the right lateral intraventricular hemorrhage (white arrow)

The patient was taken emergently to the operating room by the neurosurgery team. The patient received cefazolin, vancomycin, cefepime, and metronidazole prior to skin incision due to the contaminated nature of the wound. A left pterional incision was made and T’d into the knife. A left temporal craniectomy was drilled around the knife. Once the craniectomy was completed, the knife was carefully removed from the cranium. An external ventricular drain was placed after an intraoperative head CT scan showed increased hemorrhage along the injury tract. The patient’s postoperative head CT scan is shown in Figure 4.

**Figure 5:**
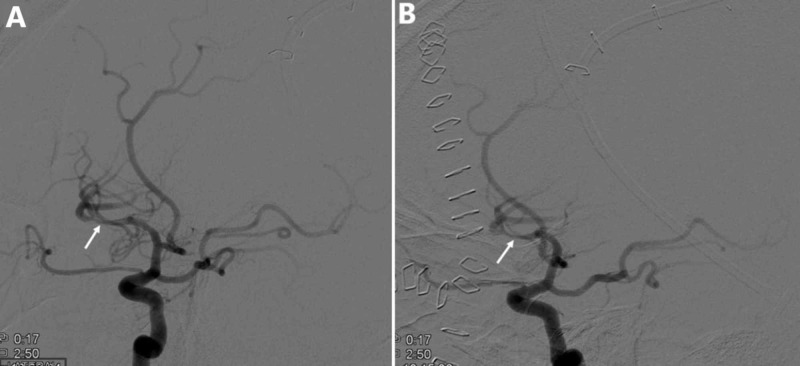
Digital subtraction angiography of the left internal carotid artery and its major intracranial branches six days postinjury. (A) Focal area of vasospasm in the left inferior division of the middle cerebral artery M2 segment before intra-arterial verapamil administration (white arrow). (B) Focal area of vasospasm with mild improvement in the left inferior division of the middle cerebral artery M2 segment after intra-arterial verapamil administration (white arrow).

The patient was following commands with all extremities on the first postoperative day. He developed hydrocephalus requiring cerebrospinal fluid (CSF) diversion, so the external ventricular drain was left in place for six days. A formal cerebral angiogram was done on postoperative day six showing a focal area of vasospasm of the left inferior division of the middle cerebral artery M2 segment (Figure 5). This was treated with intra-arterial verapamil with mild improvement on completion angiogram. 

The patient was febrile early in his postoperative course and therefore completed 12 days of broad spectrum antibiotics. Two sets of blood cultures were negative. He completed seven days of levetiracetam for early seizure prophylaxis. He was discharged to an inpatient rehabilitation facility 13 days postinjury. He was discharged to home from rehab on postinjury day 21. The patient has not followed up with neurosurgery since his discharge. However, he returned to the ER for suture removal almost one year after the injury and did not appear to have neurologic sequelae. Based on chart review of this visit, the patient’s Glasgow Outcome Score would be five.

## Discussion

Background

The yearly incidence of transcranial stabbings is largely unknown. Young men are reported to be the most common demographic affected [1]. PBIs may result from high- or low-velocity mechanisms. The numerical cutoff between low- and high-velocity projectiles is variably defined in the literature [2-4]. Perhaps more important than defining the exact number at which a low-velocity injury becomes high velocity is recognizing the disparate injury patterns with each mechanism. Low-velocity projectiles, such as knives or low-caliber hand gun bullets, do not impart the concussive force and thermal energy associated with high-velocity projectiles, such as rifle bullets. Tissue damage is thus more contained to the projectile’s intracranial tract. Therefore, the location of the lesion in low-velocity PBIs is paramount in predicting mortality and outcomes. For example, in a series of 10 patients who suffered temporal fossa stab wounds, all patients who experienced pontine or basilar artery injuries died [5]. Low-velocity projectiles are most likely to enter the skull where it is the thinnest. Common places for intracranial penetration include the orbital roof, cribriform plate, and temporal squama [6]. Some authors differentiate between stab wounds that penetrate the cranium and those that access natural orifices such as the orbit. It is unclear if natural orifice penetrating injuries result in distinct intraparenchymal trauma. However, projectiles that traverse air sinuses are associated with increased infectious complications.

The patient in this report presented with several characteristics associated with poor outcomes and low survival rates. Adults with bihemispheric craniocerebral GSWs have higher mortality (82%) than patients with one hemisphere involved (62%) [7]. Involvement of midline structures such as the thalami or basal ganglia on CT imaging is also associated with poor outcomes [8]. The anatomy of the injury tract warrants particular attention. As Haworth and de Villiers noted, blades that are angled downward in the temporal fossa may be stopped by the petrous ridge before encountering deep structures [5]. However, a downward and posterior blow may drive the blade into the brain stem or basilar artery. Similarly, a swinging blow which strikes low on the pterion may be directed anteriorly and injure the cavernous sinus. In this report, the knife was likely directed with an upward, slightly posterior force through the superior half of the left temporal lobe such that the pterion, petrous ridge, or sphenoid bone could not block or direct its path. This could partly explain the extent of the blade’s intracranial penetration.

Survivorship is higher in patients with intracranial stab wounds compared to high-velocity injuries. In two series of patients with stab wounds to the brain, the combined mortality was 23% [5, 9]. A more contemporary study reported even lower mortality (11%) in a series of 66 patients with transcranial stab wounds [10]. However, stab wounds penetrating the orbit are associated with mortality of up to 30% in at least one series [11]. In contrast, overall mortality from GSWs to the head can be as high as 91% [12]. Mortality among patients who survive long enough to be admitted to the hospital ranged from 49% to 70% in three studies [12-14]. Management of intracranial GSWs is beyond the scope of this report. However, many of the management principles for high-velocity PBIs may apply to low-velocity PBIs.

Management

Initial assessment of the patient according to advanced trauma life support protocol is critical. Patients should be intubated expeditiously if providers are concerned they may not be able to protect their airway. Several signs have been shown to associate with poor outcomes in PBIs, including initial GCS score, abnormal pupillary responses, hypotension, coagulopathy, and advanced age [1, 8]. Once the patient has been stabilized, noncontrasted head CT scan should be performed for intracranial stab wounds. Plain radiographs may be done if a CT scan is unavailable or if it is low quality secondary to beam artifact. Early CT angiography (within six hours of presentation) is useful in identifying initial intracranial vascular injuries, but it can be limited due to interference from the retained projectile [10]. Additionally, a negative CT angiographic exam does not preclude subsequent development of vascular complications. MRI has a limited role in patients with retained stab wounds as the foreign objects almost always contain metal. Patients with retained foreign objects penetrating the dura and brain parenchyma will nearly always need surgery to remove the object.

The operative plan for these patients is largely dependent on the entry and termination points of the foreign object. Major goals of any operation include debridement of soft tissue and bone, achieving hemostasis, dural closure, and removal of the foreign object under direct visualization. Multidisciplinary approaches may be necessary with head and neck surgeons if facial structures are in the projectile’s path. Skin incision and craniotomy approaches are at the surgeon’s discretion. Wide exposure is necessary as vessels and tissues that were under the tamponade effect may hemorrhage once the object is removed. A rocking, back and forth movement should be avoided when removing the object to avoid further injuries. One paper suggests applying vice grips to the exposed portion of the object and using a small hammer to carefully strike the grips to facilitate in-line removal [10].

Patients with transcranial stab wounds are at heightened risk for delayed vascular complications, such as traumatic intracranial aneurysm, pseudoaneurysm, vasospasm, intravascular thrombosis, and carotid-cavernous fistula. The reported prevalence of vascular complications following both low- and high-velocity PBIs is as high as 50% [15]. Taylor and Peter reported a 23% vascular complication rate in a series of 66 patients with transcranial stab wounds [10]. The consequences of traumatic aneurysm rupture can be devastating. In one series, 36% of patients diagnosed with traumatic aneurysms on angiography after transcranial stabbings died from aneurysm rupture [16]. Formal angiography is an important diagnostic and therapeutic tool. Initial CT angiography may not reveal vascular lesions due to interference from the retained projectile, and vascular complications may present weeks to months after the injury. For this reason, some authors have advocated digital subtraction angiography by the second to third week following a PBI [16-17].

Most transcranial stabbings occur with contaminated projectiles, placing patients at heightened risk for central nervous system infections. In a series of patients who suffered intracranial trauma from wooden projectiles, 48% developed a brain abscess [18]. Broad spectrum antibiotics which cover Gram positive, Gram negative, and anerobic bacteria are recommended and should ideally be administered before operative intervention. The duration of antibiotics for prophylactic coverage is debated. Some authors recommend up to 14 days of prophylaxis [19]. If an infection is suspected or confirmed, the duration of antibiotic therapy may lengthen depending on culture results. CSF leaks may increase the risk for infection, and a water tight seal with suture or graft material is necessary. Additionally, up to 50% of patients with PBIs may develop post-traumatic seizure disorders, and 10% of these patients may experience one in the early postinjury period [20]. Therefore, early seizure prophylaxis is recommended for seven days postinjury. Seizure prophylaxis beyond seven days has not been shown to be effective in limiting the development of post-traumatic seizure disorders.

## Conclusions

Here, we have shown that survival and good functional outcome are possible even after a bihemispheric stab wound involving subcortical structures. Low-velocity PBIs pose significant treatment challenges to the medical team caring for this relatively uncommon cohort of patients. Noncontrasted head CT scan and CT angiography are recommended for initial evaluation. Operative intervention is necessary to remove retained projectiles. Digital subtraction angiography is recommended to assess for delayed traumatic vascular complications. Antibiotics and anti-epileptic medications are recommended for prophylaxis in the postoperative period. 
